# An adaptive optimal viewing angle determination algorithm for TEVAR operation

**DOI:** 10.1186/s12880-021-00676-3

**Published:** 2021-10-02

**Authors:** Weiya Sun, Guanyu Yang, Yang Chen, Huazhong Shu

**Affiliations:** 1 Laboratory of Image Science and Technology, Key Laboratory of Computer Network and Information Integration, School of Computer Science and Engineering, Southeast University, Nanjing, 210096 China; 2 Centre de Recherche en Information BioMdicale Sino-Franais (CRIBs), Nanjing, China

**Keywords:** Computed tomography angiography, X-ray, Low-dose, Optimum viewing angle, Aortic dissection

## Abstract

**Background:**

The determination of the right x-ray angiography viewing angle is an important issue during the treatment of thoracic endovascular aortic repair (TEVAR). An inaccurate projection angle (manually determined today by the physicians according to their personal experience) may affect the placement of the stent and cause vascular occlusion or endoleak.

**Methods:**

Based on the acquisition of a computed tomography angiography (CTA) image before TEVAR, an adaptive optimization algorithm is proposed to determine the optimal viewing angle of the angiogram automatically. This optimal view aims at avoiding any overlapping between the left common carotid artery and the left subclavian artery. Moreover, the proposed optimal procedure exploits the patient-specific morphology to adaptively reduce the potential foreshortening effect.

**Results:**

Experimental results conducted on thirty-five patients demonstrate that the optimal angiographic viewing angle based on the proposed method has no significant difference when compared with the expert practice (*p* = 0.0678).

**Conclusion:**

We propose a method that utilizes the CTA image acquired before TEVAR to automatically calculate the optimal C-arm angle. This method has the potential to assist surgeons during their interventional procedure by providing a shorter procedure time, less radiation exposure, and less contrast injection.

## Background

Type B aortic dissection (TBAD) is an acute cardiovascular disease with high mortality and disability rates [1–3]. Medical treatment is routinely used in the clinic to treat the uncomplicated aortic dissection. However, for the complicated TBAD, the thoracic endovascular aortic repair (TEVAR) is an alternative and effective tool compared to open surgery [4–6]. Since the stent placement is mainly guided by digital subtraction angiography (DSA), an inaccurate viewing angle may lead to 3D vascular structures overlap in the projected 2D images. Therefore, the viewing angle of the C-arm plays an important role in placing the stent grafts.

During the process of TEVAR, physicians manually determine the angiographic viewing angles according to their personal experiences in order to display the whole aortic arch while eliminating any overlap of branches. This task is subjective and may not only be time-consuming for young physicians but also increase the patient’s intake of X-ray and contrast agent. Furthermore, the X-ray angiographic images with improper viewing angles lose much aortic topological information [7, 8], thus, affect the accuracy of the stent placement and lead to some later complications such as aortic rupture or retrograde dissection [9]. The current clinical imaging angle selection requires the participation of expert cardiologists, hence an additional burden in the clinical setting. Therefore, the automatic selection of angiography viewing angle is extremely important and clinically required.

The issue of automatic optimum viewing angle determination has attracted many researchers’ attention because of its major clinical implications. Dumay et al. [10] assumed that the interested vessel is a cylinder and selected two projection images of different angles. Then, the optimal viewing angle was obtained through the geometry relationship of corresponding vectors. Nevertheless, this method is only suitable for slightly-curved vessels and requires at least two different angles of projection images. It fails in case of large and extended curvature. Moreover, obtaining projection images at different angles increase the radiation dose. Considering the intake of X-ray and contrast agent, a series of studies used CTA data to obtain the optimal angle in the coronary artery [11–16]. The vessel vector was represented by the line between two adjacent points on the centerline of the vessel of interest. Chen et al. [17] used a predefined threshold to calculate the minimum vascular projection foreshortening rate. The optimal angiographic angle was obtained by refining the overlapping rate. Considering that the aortic branches are much larger than the coronary arteries, the method applied in coronary arteries is not suitable for the aorta. The imaging angle automatically obtained through mathematically minimum methods does not fit the clinical scenario in the aorta. The optimal angle obtained by adding the constraint design of the realistic clinical scenario is more suitable for the clinic.

In order to solve the aforementioned drawbacks, we proposed an adaptive optimization method that automatically determines the angiography viewing angle for the TEVAR operation. The projection foreshortening rate (PFR) is used to obtain a full display of the aortic arch, and the projection overlapping rate (POR) is used to avoid the overlap of branches on the aortic arch. By combining an empirical regularization term, the optimal angle can be automatically obtained before TEVAR, which ensures doctors a better imaging angle to place the stent more accurately. The contributions of this work are summarized as follows:

1. To our knowledge, our solution is the first attempt based on pre-operative CTA images for providing an automatically and reliably optimal imaging angle view for TEVAR.
2. The optimum viewing angle is obtained by optimizing the display of the aortic arch without overlapping branches instead of minimizing the POR and PFR.
3. Combined with the clinical scenario of the aorta, the proposed method adaptively provides junior doctors with an optimal imaging angle pre-operation.
4. This method has the potential to optimize the planning time, improve the accuracy of the stent placement and reduce the radiation exposure during TEVAR.

## Methods

**Fig. 1:**
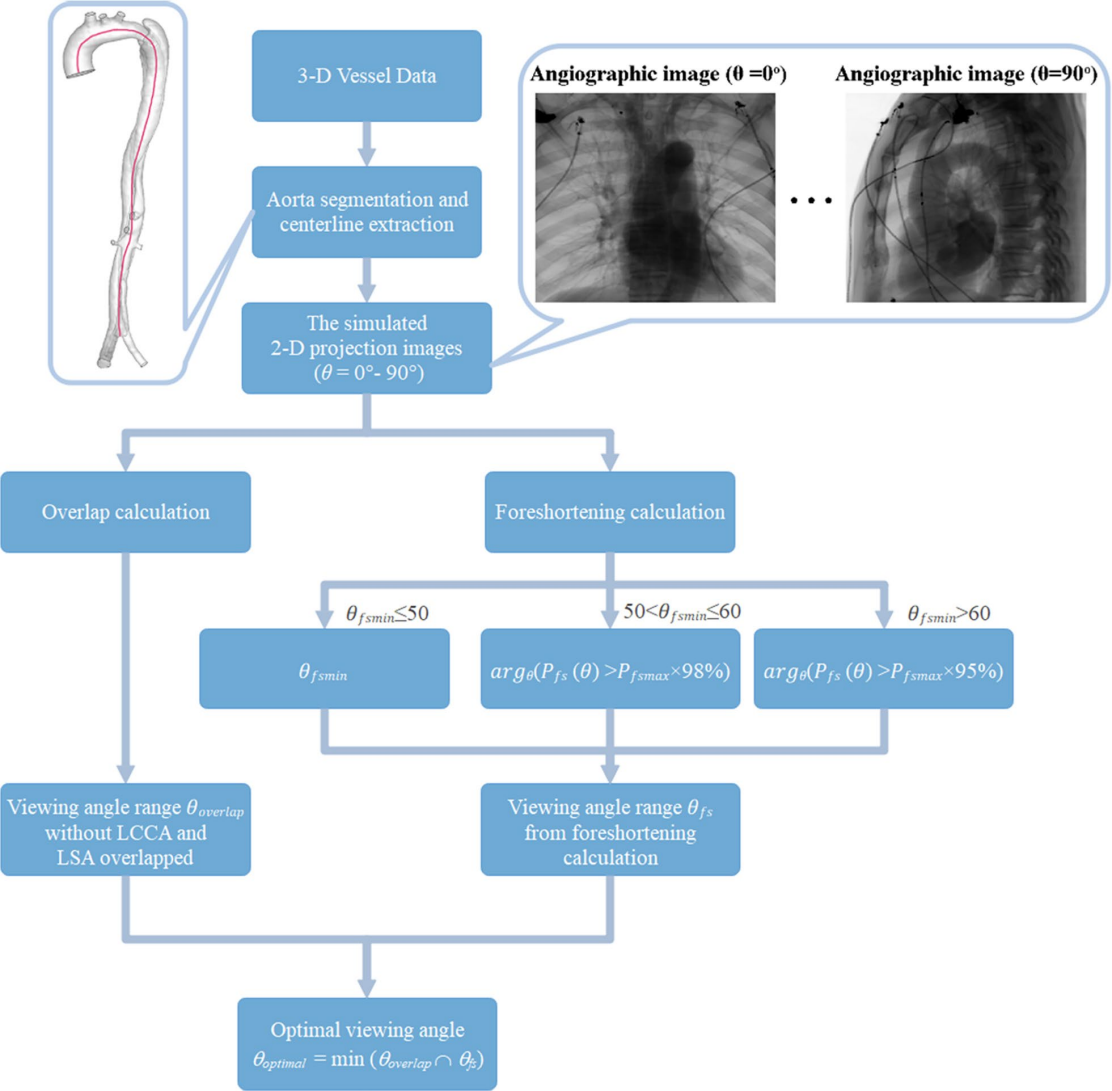
Flowchart of the proposed solution

Patients with aortic dissection before TEVAR usually have a CTA examination. Based on the CTA data, the aorta is segmented and the centerline is obtained. CTA data are then used to simulate angiograms. The amount of foreshortening and vessel overlap for the aortic arch can be calculated. Without the left common carotid artery (LCCA) and left subclavian artery (LSA) overlapped, an adaptive optimization approach is proposed to determine the viewing angle before the operation automatically. Figure 1 shows the flow chart of the proposed algorithm.

### Patients and image acquisition

Our institutional review board approved this study. We focus on the TBAD patients who needed the interventional operation from August 2019 to December 2020: 63 patients in the acute phase underwent TEVAR identified from 107 type B aortic dissection patients. Detailed in-hospital information is given in Table 1. Among these 63 patients, we excluded the patients with procedure unsuccess (*n* = 2), patients who died in the hospital (*n* = 3), patients submitted to the adjuvant procedure (*n* = 10), patients who lost DSA or CTA data before intervention (*n* = 10) and TEVAR covered left subclavian artery (*n* = 3) from the dataset. The final patient set consists of 35 patients. The basic characteristics of patients are summarized in Table 2. Each CT angiography dataset is acquired before stent-graft implantation via a dual-source CT scanner (SOMATOM Definition Flash, SIEMENS, Germany) and the intervention is performed under the DSA Artis Zee (SIEMENS, Germany). Two experts (cardiovascular radiologists) work together to manually acquire the viewing angle of the DSA during TEVAR for all the patients. During the CTA, the patient lies on his back on the table with his upper limbs raised vertically above the head and is placed in a supine position during the intervention with his arms down naturally at his sides.

**Table 1:** In-hospital outcome

Characteristic	No. (%)
Acute phase	63
Procedure unsuccess	2 (3.2%)
In-hospital mortality	3 (4.8%)
Aorta related mortality	1 (1.6%)
Non aorta related mortality	2 (3.2%)
Adjuvant endovascular procedures^#^	10 (15.9%)
Patients with insufficient data^&^	10 (15.9%)
Covered left subclavian artery	3 (4.8%)

^#^Adjuvant endovascular procedure: chimney, fenestration, cervical artery bypass, visceral artery or iliac artery stenting

^&^10 patients lack of CTA or DSA data before TEVAR

**Table 2:** Basic characteristics of the patients

Patients characteristics	Value
Age, year	53.9 (12.6)
Male, %	31 [88.6]
Hypertension	30 [85.7]
Smoking	14 [40]
Diabetes mellitus	10 [28.6]
Coronary heart disease	1 [2.9]

Continuous data are presented as mean (standard deviation); and discontinuous data are presented as amount [percentage]

### Projection overlapping rate

**Fig. 2:**
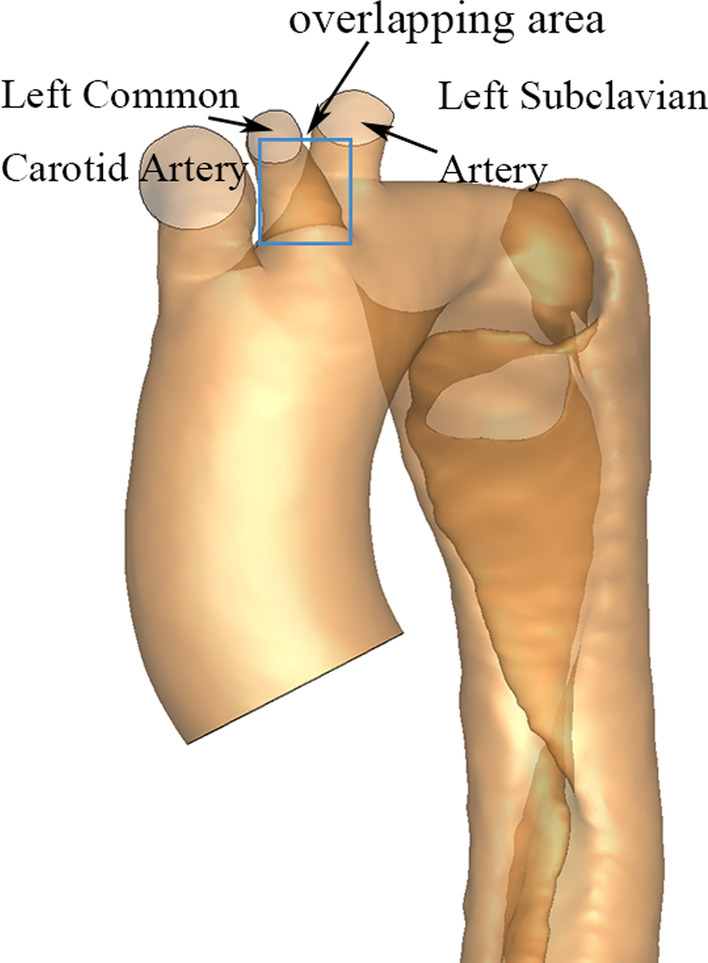
An example of vessel overlap. The overlapping area in the projection image for the left common carotid artery and the left subclavian artery

The 2D projection principle in X-ray imaging leads to the superimposition of all tissues and, in our case, may lead to an overlap of the branches connected to the aortic arch (i.e. LCCA and LSA). This overlap can affect the placement of the stent in patients with type B aortic dissection. The blue square in Fig. 2 shows an example of such overlap where it is difficult to distinguish the boundaries of each branch. Improper placement of stents covering the branches of the aortic arch can cause organ ischemia. The optimum viewing angle should therefore be estimated by minimizing the overlapping rate of the LCCA and LSA.

Let *S*_*LCCA*_(*θ*) and *S*_*LSA*_(*θ*) represent the LCCA and LSA segments in the projection image at angle *θ* respectively, the POR *P*_*overlap*_ can be calculated by counting the pixels in the projection image as:

1 $$P_{overlap} (\theta ) = \frac{{NP\left[ {S_{LCCA} (\theta ) \cap S_{LSA} (\theta )} \right]}}{{NP\left[ {S_{LCCA} (\theta )} \right] + NP\left[ {S_{LSA} (\theta )} \right]}}$$

where *NP*(*A*) denotes the number of pixels of *A*.

### Projection foreshortening rate

**Fig. 3:**
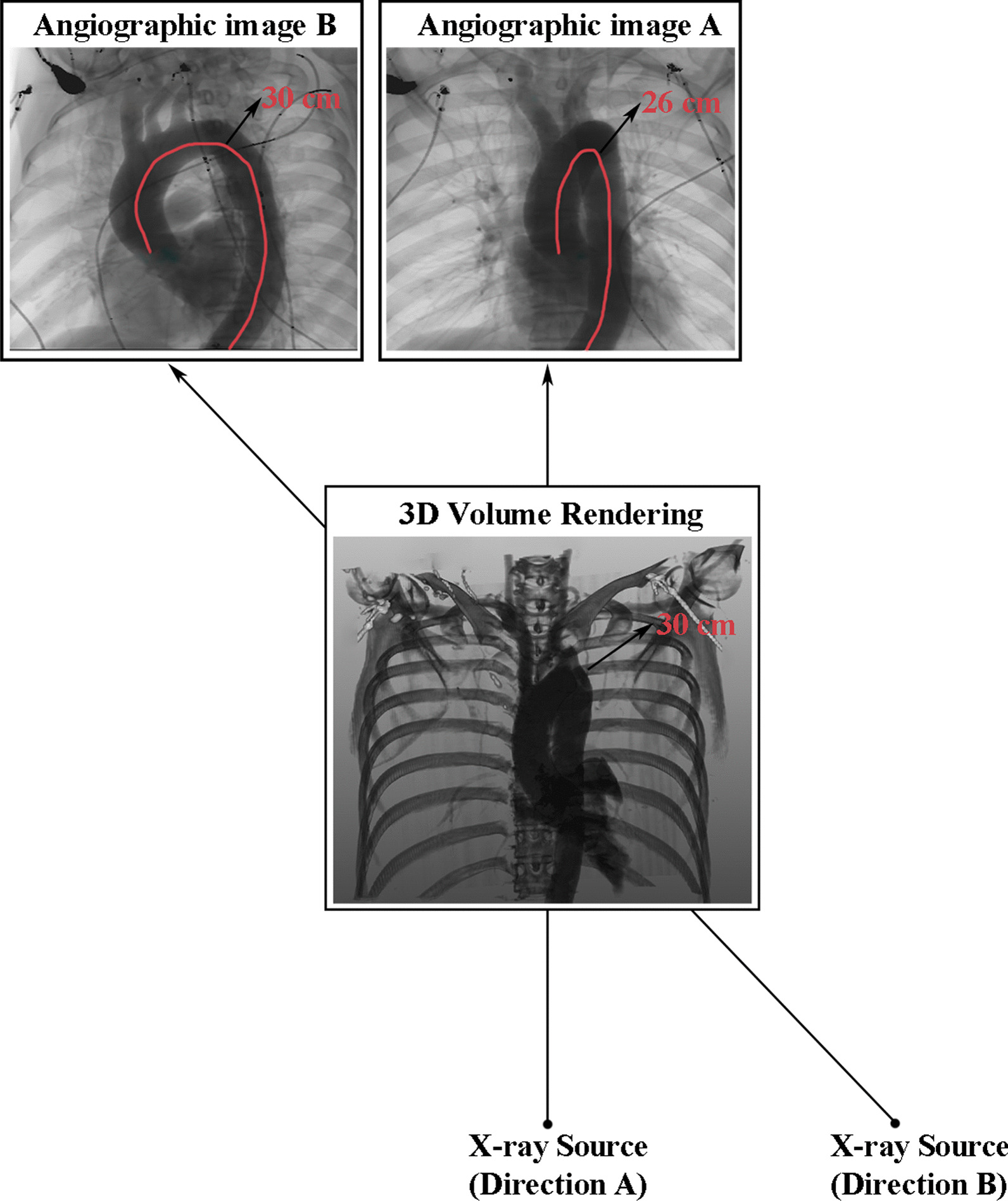
Angiographic images obtained from different X-ray directions. The true 3D length of the aorta is 30 cm. In the angiographic image A (i.e. direction A), important overlaps can be observed, associated with a significant foreshortening (the length of the aorta is 26 cm). The angiographic image B (i.e. direction B, perpendicular to the aortic arch plane), there is no overlapping nor foreshortening (the length of the aorta is 30 cm)

In the 2D angiographic image, when the X-ray direction is not perpendicular to the aortic arch plane, the projection of the length of the aorta in the two-dimensional image is shorter than the length in 3D. In Fig. 3, we simulate the angiographic image by computing the X-ray projections from different directions and measure the length of the aorta. It is estimated in image A to 26 cm, much shorter than the actual length (30 cm) in 3D. In image B, the aortic arch is fully displayed and does not have any foreshortening. Direction B can be almost perpendicular to the aortic arch plane. It is difficult to determine the best placement of the stent when the aortic arch overlap. Displaying the aortic arch as completely as possible in 2D angiography permits the physicians to observe the aortic structure information clearly, thus facilitate the accurate placement of the stent. The PFR *P*_*fs*_ of the aortic arch can be calculated by the ratio of the centerline length after projection under angle *θ* to the centerline length in CTA:

2 $$P_{fs} (\theta ) = \frac{{\int_{0}^{{T_{1} \left( \theta \right)}} {\left| {\overrightarrow {C} (t) \cdot \overrightarrow {P} } \right|dt} }}{{\int_{0}^{{T_{2} }} {\left| {\overrightarrow {C} (t)} \right|dt} }}$$

where $\overrightarrow {P}$ represents the projection vector of x-ray, $\overrightarrow {C} (t)$ is the centerline of the aorta in 3D, and *T*_1_(*θ*) represents the total length of the centerline obtained at angle *θ* in 2-D projection and *T*_2_ represents the total length of the centerline in 3-D.

### Standard algorithm

Minimizing the foreshortening rate and the overlapping rate can be expressed as solving the standard optimization problem:

3 $$\theta_{stanard} = \mathop {{\text{arg}}}\limits_{\theta } \min (P_{overlap} (\theta ) + P_{fs} (\theta ))$$

However such approach has two drawbacks here: since the sum of POR and PFR is minimized, there is no guarantee that the optimum angle will suppress any overlapping between LCCA and LSA. In addition, the morphology of the aorta is not taken into account. Therefore it seems more meaningful to adopt a two steps method by looking for angles eliminating the superimpositions and then by selecting among them the optimal view satisfying the PFR *P*_*fs*_ criteria.

### Adaptive algorithm

The detail of our algorithm to obtain the optimal view can be described as follows:

**Step 1:** The aorta in the CTA data is segmented and the centerline is obtained by the method reported in Ref. [18].

**Step 2:** Angiographic images are simulated for angle *θ* varying from 0 to 90 degrees of the C-arm based on the 3D vascular structure.

**Step 3:** Calculate the POR between LCCA and LSA by Eq. (1), and denote *θ*_*no-overlap*_ the set of angles which lead to *P*_*overlap*_(*θ*) = 0, that means, there is no overlap between LCCA and LSA.

**Step 4:** Calculate the PFR by Equation (2) for each given value of *θ*, and we choose the angle *θ*_*fsmin*_ which reaches the maximal value of foreshortening *P*_*fs*_ denoted hereafter by *P*_*fsmax*_. If the angle *θ*_*fsmin*_ is too large, an adaptive strategy is adopted to meet the clinical requirement. To achieve this goal, we define:

4 $$\theta_{fs} = \left\{ {\begin{array}{*{20}c} {\theta_{{fsm{\text{in}}}} ,\theta_{{fsm{\text{in}}}} \le 50} \\ {\mathop {\arg }\limits_{\theta } (P_{fs} (\theta ) > P_{fs\max } \times 98\% ),\;50 < \theta_{fs\min } \le 60} \\ {\mathop {\arg }\limits_{\theta } (P_{fs} (\theta ) > P_{fs\max } \times 95\% ),\;\theta_{fs\min } > 60} \\ \end{array} } \right.$$

**Step 5:** Calculate the intersection of *θ*_*no-overlap*_ and *θ*_*fs*_, the minimal value of this interval is chosen as the optimal view angle *θ*_*optimal*_.

## Results

Qualitative and quantitative analyses are performed on the dataset described above and comparisons are carried out between the standard approach, the proposed adaptive method using the ground truth of C-arm angles set by the cardiovascular radiologist during TEVAR.

### Quantitative analysis

The statistical analysis is performed using Prism 7.0 (GraphPad Software Inc, San Diego, Calif). If the assumption of normality is met, statistically significant differences for the viewing angle result obtained from different algorithms are evaluated using the T-test. However, if the assumption of normality is not met, a Mann–Whitney U test was performed. Values of *P* < 0.05 are considered statistically significant.

**Fig. 4:**
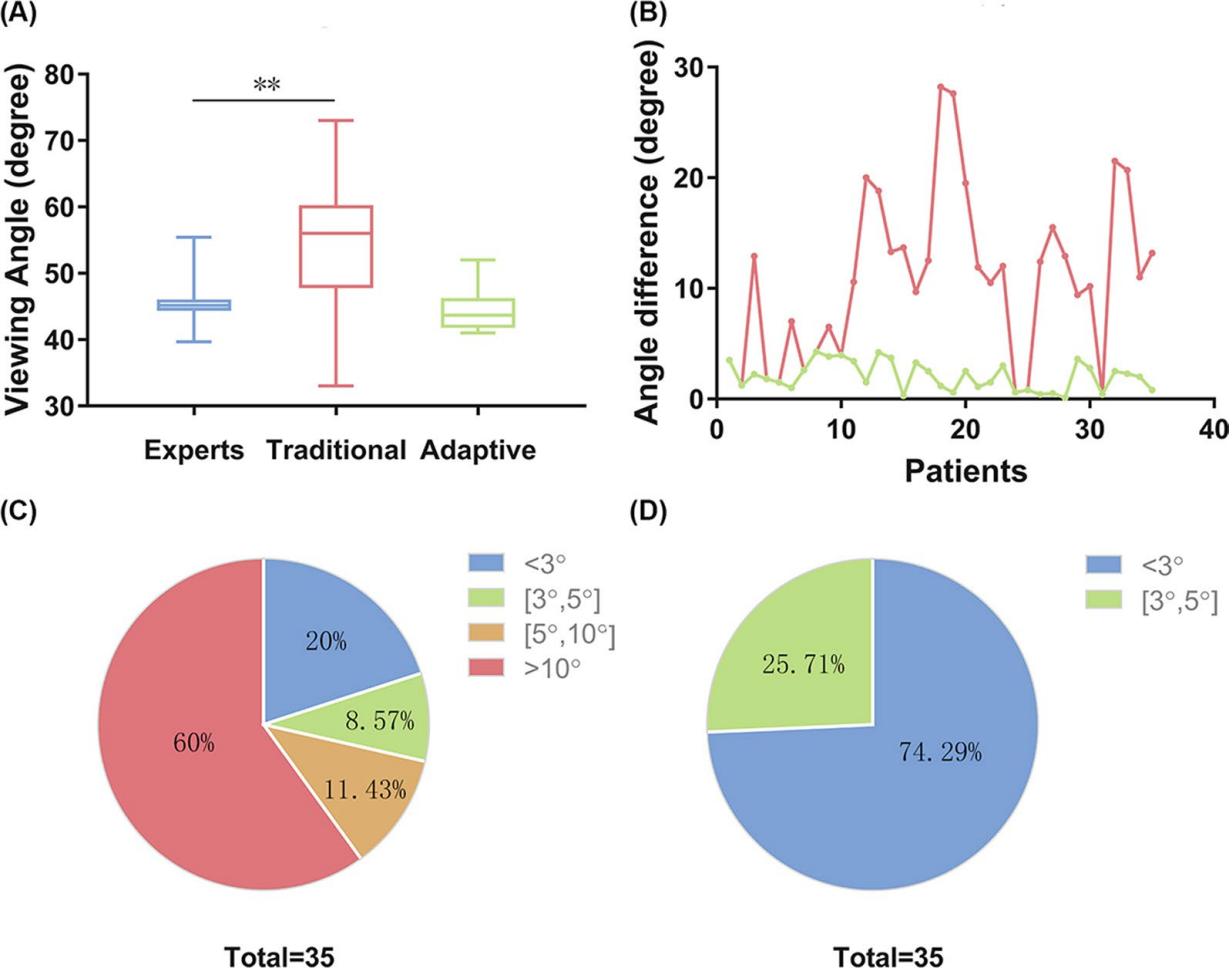
**a** Boxplot of chosen angles by the experts, standard and adaptive method. **b** Line charts of angle difference between experts and automatic methods. The red line depicts the absolute angle difference between the expert setting and the result of the standard method. The green line shows the absolute difference between experts’ positioning and angle obtained by means of the adaptive method. **c** The proportions of angle difference between the results of experts and the standard method. **d** The proportions of angle difference between the results of experts and the adaptive method

Figure 4a shows the optimal viewing angles obtained for 35 aortic dissection patients. The means and standard deviations are respectively 54.67 ± 1.645 (standard approach), 44.55 ± 0.5014 (adaptive method) and 45.79 ± 0.4474 (experts). The differences between the standard algorithm and experts are significant (*p* < 0.0001), no significant difference is observed between the experts and the adaptive results (*p* = 0.0678).

Figure 4b summarizes these results. The red line depicts the absolute angle difference between experts and the standard minimization approach and the green line is the absolute difference between our proposed algorithm and expert settings. It can be seen that the adaptive viewing angle optimization has less error than the standard one. The distributions of the angle differences between the three frames are presented in Fig. 4c and d, respectively. While in the former case (4C) only twenty percent of differences are less than three degrees, when looking at the adaptive versus experts angle setting, the differences are less than five degrees, and most of them less than three degrees.

### Qualitative analysis

**Fig. 5:**
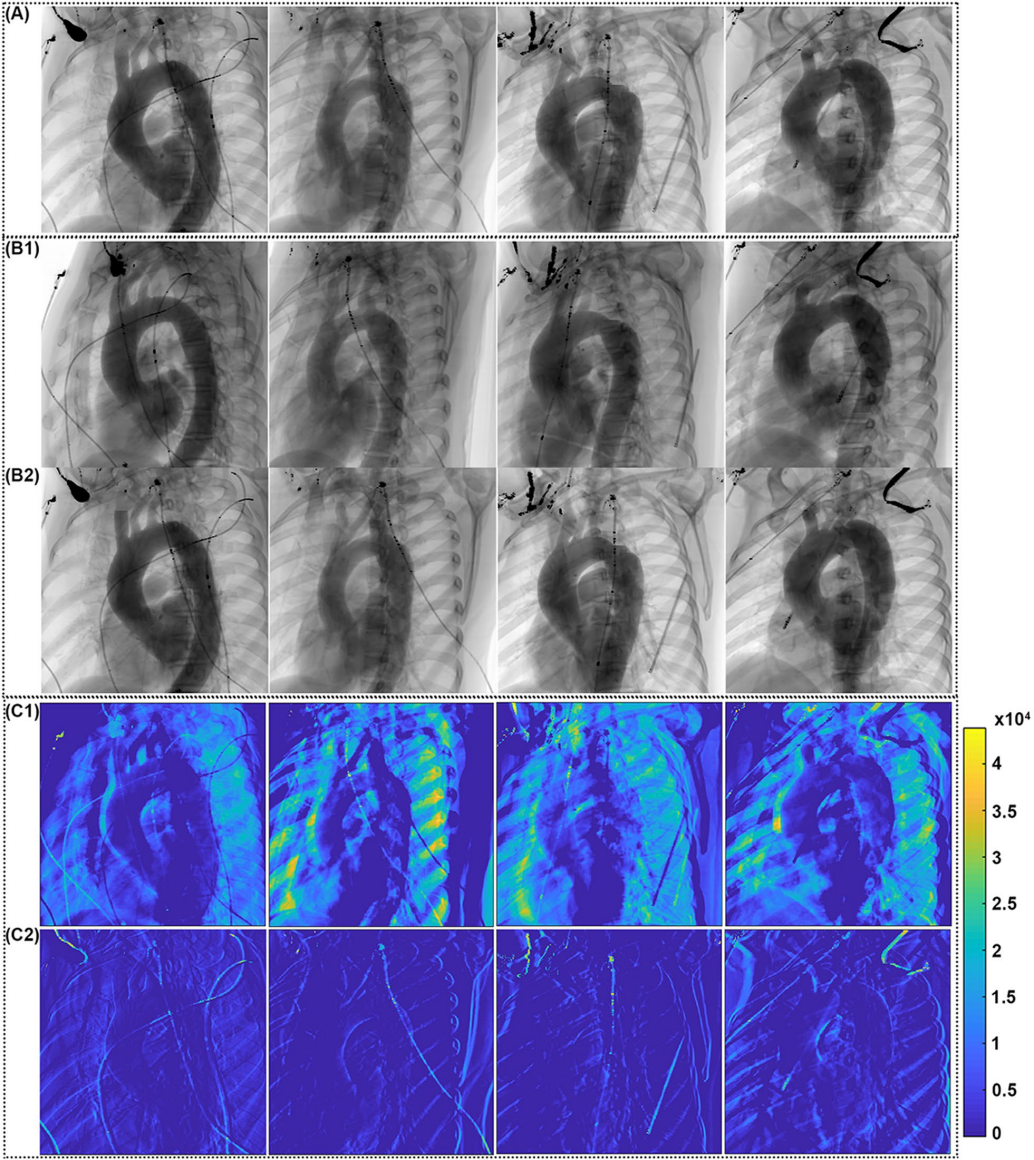
Simulation of CTA projections using the expert angle choice and the automatic angle determination: **a** projection result based on the experts. **b1** projection provided using the optimal view resulting from the standard method. (B2) the same based on the adaptive algorithm. **c1** and **c2** absolute difference images between the ground truth A and the images **b1** and **b2** respectively in color scale (This color code is the error value of the grayscale)

In order to evaluate the performance of the proposed method, the clinical CTA is used to simulate a 2D angiographic imaging system. Figure 5 shows the simulated angiographic view by experts (first row) used during the intervention, the standard (second row) and adaptive algorithm (third row) results obtained on four patients. In Fig. 5C1 and C2 display the absolute difference image between the ground truth projection and the projections obtained through the standard and adaptive algorithms respectively. A color image coding is adopted in order to visually enhance the differences observed. They are major in 5C1 while, according to the blue color dominance in 5C2, they are close to zero. The aortic arch is displayed without LCCA and LSA overlapping both in the simulated projection based on expert setting and on the angle estimated by our approach.

## Discussion

As an option for patients with type B aortic dissection, TEVAR has been proved a sound technique to replace open surgery [19, 20]. However, its efficacy depends on whether the proximal entry tear is completely covered or not. Therefore appropriate stent selection and deployment techniques are required. In addition, undesirable results such as occlusion or endoleak could result from a suboptimal stent deployment caused by an unreliable viewing angle [21, 22]. During the stent placement, the optimal viewing angle is subjectively selected by adjusting the rotation angle of the X-ray gantry. This “trial-and-error” approach increases the dose of contrast agent and radiation exposure to the doctors and patients. Besides, due to the patient-specific aortic patterns, there is no guarantee that the angle chosen by the less experienced doctor could visualize the aorta optimally during the stent deployment [23, 24]. Therefore, computer-assisted selection of the optimal viewing angle is of great significance to support junior doctors during TEVAR, especially with the increasing complexity of interventional treatment for aortic dissection.

In some cases, the identification of optimal viewing angles based on 2D angiographic projections is extremely challenging. Since the reconstruction of the whole aorta in angiographic acquisitions is highly time-consuming and needs a high quality angiographic image system [7], it appears difficult to apply this approach in routine clinical practice. In order to propose an effective and practical solution, we only need to reconstruct the thoracic aorta using the CTA data acquired before the intervention. Once the 3D reconstruction has been completed, the information can be used to plan the intervention-related image acquisition, which will benefit the patients who need repeated radiation exposure.

The projection overlapping rate and projection foreshortening rate are two keys factors in the optimum angle determination. The former represents the overlap of the branches on the aortic arch, which is clinically used to avoid any stent covering the branches and to guide the stent placement in the endovascular procedure. As a functional estimation, the latter indicates whether the aortic arch displays the anatomical information clearly at this viewing angle. The proposed method utilizes both the PFR and POR. Instead of considering a joint minimization with the PFR and POR terms, we have shown how we can first deal with branch overlaps and then look among the solutions the optimal view minimizing any possible foreshortening. No significant difference is observed between our results and the C-arm angle setting given by the experts during TEVAR.

The determination of C-arm angle is necessary for patients who require endovascular surgery. The clinically used “trial-and-error” approach, although efficient, increases the contrast agent dose and radiation exposure to the doctors and patients. The guiding principle of radiation safety (i.e. ALARA for “as low as reasonably achievable”) recommends reducing in any way the radiation even if the gain is small. Compared with the “trial-and-error” approach, our algorithm only makes use the CTA data acquired before TEVAR to get the optimal viewing angle. The results are very close to the angle chosen by the experts and no statistically significant difference is observed. Therefore, the C-arm angle can directly use the result obtained by our algorithm before the intervention. After the contrast injection, if the angle is suitable, the stent can be directly implanted. Even if the angle needs to be fine-tuned, the slight error will reduce the radiation time compared to the previous angle adjustment from zero. Our future work will focus on the overall benefit of our approach in terms of radiation amount and contrast usage by either experts or junior physicians.

## Conclusion

In this paper, an adaptive optimization algorithm based on CTA data acquired before TEVAR is proposed to determine the clinically best C-arm angle to use during the intervention. The optimal viewing angle estimated using this method does not show a significant difference with expert settings. Hence, this automatic method has the potential to assist junior doctors while providing shorter procedure time, less radiation exposure and contrast injection.

## Data Availability

The datasets used or analyzed during the current study is available from the corresponding author on reasonable request.
